# 560G>A (rs4986782) (R187Q) Single Nucleotide Polymorphism in Arylamine N-Acetyltransferase 1 Increases Affinity for the Aromatic Amine Carcinogens 4-Aminobiphenyl and N-Hydroxy-4-Aminobiphenyl: Implications for Cancer Risk Assessment

**DOI:** 10.3389/fphar.2022.820082

**Published:** 2022-02-22

**Authors:** Mark A. Doll, David W. Hein

**Affiliations:** Department of Pharmacology and Toxicology, University of Louisville School of Medicine, Louisville, KY, United States

**Keywords:** arylamine N-acetyltransferase 1, single nucelotide polymorphisms, 4-aminobiphenyl, N-hydroxy-4-aminobiphenyl, N-acetylation, O-acetylation

## Abstract

Human arylamine N-acetyltransferase 1 (NAT1) catalyzes the N-acetylation of arylamine carcinogens such as 4-aminobiphenyl (ABP), and following N-hydroxylation, the O-acetylation of N-hydroxy-arylamine carcinogens such as N-hydroxy-ABP (N-OH-ABP). Genetic polymorphisms in NAT1 are linked to cancer susceptibility following exposures. The effects of individual single nucleotide polymorphisms (SNPs) in the NAT1 coding exon on Michaelis-Menten kinetic constants was assessed for ABP N-acetyltransferase and N-OH-ABP O-acetyltransferase activity following transfection of human NAT1 into COS-1 cells (SV40-transformed African green monkey kidney cells). NAT1 coding region SNPs 97C > T (rs56318881) (R33stop), 190C > T (rs56379106) (R64W), 559C > T (rs5030839) (R187stop) and 752A > T (rs56172717) (D251V) reduced ABP N- acetyltransferase and N-OH-ABP O-acetyltransferase activity below detection. 21T > G (rs4986992) (synonymous), 402T > C (rs146727732) (synonymous), 445G > A (rs4987076) (V149I), 613A > G (rs72554609) (M205V) and 640T > G (rs4986783) (S241A) did not significantly affect Vmax for ABP N-acetyltransferase or N-OH-ABP O-acetyltransferase. 781G > A (rs72554610) (E261K), and 787A > G (rs72554611) (I263V) slightly reduced ABP N-acetyltransferase and N-OH-ABP O-acetyltransferase activities whereas 560G > A (rs4986782) (R187Q) substantially and significantly reduced them. 560G > A (rs4986782) (R187Q) significantly reduced the apparent Km for ABP and N-OH-ABP a finding that was not observed with any of the other NAT1 SNPs tested. These findings suggest that the role of the 560G > A (rs4986782) (R187Q) SNP cancer risk assessment may be modified by exposure level to aromatic amine carcinogens such as ABP.

## Introduction

Human occupational exposures to 4-aminobiphenyl (ABP) led to excess incidence of urinary bladder cancer with sufficient evidence for listing as a Group 1 carcinogen (IARC, 1987). ABP also is carcinogenic in animal models including liver, intestine, mammary gland, and angiosarcoma (NTP, 2021). Subsequent human epidemiological investigations have focused on the elevated cancer incidence in cigarette smokers, particularly in the urinary bladder (NTP, 2021). In addition to both mainstream and sidestream cigarette smoke, ABP is a byproduct in the synthesis of numerous chemicals and color additives (NTP, 2021). DNA adducts following exposure to ABP serve as a biomarker of internal exposure and possibly carcinogenesis in human tissues (NTP, 2021).

Arylamine carcinogens such as ABP are *N*-hydroxylated by cytochrome P450 to N-hydroxy-ABP (N-OH-ABP) followed by O-acetylation catalyzed by arylamine N-acetyltransferase 1 (NAT1) and 2 (NAT2) to N-acetoxy-ABP which is highly unstable resulting in DNA adducts. DNA adducts not repaired can result in mutations leading to cancer (Wang et al., 2019; NTP, 2021). NAT1 catalyzes ABP N-acetyltransferase and N-OH-ABP O-acetyltransferase activities (Hein et al., 1993; Millner et al., 2012; Zhou et al., 2013; Leggett et al., 2021; Leggett et al., 2022; Hein et al., 2022).

Many arylamines and alkylanilines undergo N- and O-acetylation catalyzed by NAT1 (Minchin et al., 1992; Hein et al., 1993; Zenser et al., 1996; Liu et al., 2007; Leggett et al., 2021). R127 and Y129 amino acids in NAT1 reduce the volume of the NAT1 binding pocket by ∼40% compared to NAT2 (Wu et al., 2007; Zhou et al., 2013). NAT1 is selective for acetylation of 4-alkylanilines due to binding to V216, which is replaced by S216 in NAT2 (Liu et al., 2007). NAT1 exhibits higher affinity than NAT2 for the O-acetylation of N-hydroxy-arylamine carcinogens such as N-OH-ABP (Hein et al., 2022).

In addition to its role in the metabolism of exogenous drugs and xenobiotics, NAT1 has additional roles in metabolism of endogenous compounds including the hydrolysis of AcCoA (Laurieri et al., 2014; Stepp et al., 2015; Stepp et al., 2019). Further studies suggest a role for NAT1 in cellular metabolism of endogenous amines (Carlisle et al., 2020).

Human NAT1 has been measured in virtually all human organs and exhibits genetic polymorphism in human populations. Associations between NAT1 genetic polymorphism with cancer risk has been reviewed (Hein et al., 2000; Agundez 2008; Butcher and Minchin, 2012; Zhang et al., 2015). Most of these focus on associations between NAT1*10 and/or NAT1*11 and cancer risk, two variant alleles that possess SNPs in the 3′-UTR region outside of the coding exon. Urinary bladder cancer is most frequently associated with NAT1 genetic polymorphism (Cascorbi et al., 2001; Yassine et al., 2012; Dhaini et al., 2018; El-Kawak et al., 2020).

NAT1*4 is the reference allele. Numerous single nucleotide polymorphisms (SNPs) are present in the NAT1 coding exon. As previously reviewed (Hein, 2009), the identity of these SNPs and their functional effects have been investigated following recombinant expression of NAT1*4 and NAT1 variant alleles in bacteria, yeast, Chinese hamster ovary, and COS-1 cells and have focused primarily on the N-acetylation of a prototype NAT1 substrates *p*-aminosalicyclic acid and *p*-aminobenzoic acid. The investigations have identified several SNPs in the NAT1 coding exon which reduce expression of NAT1 catalytic activity and protein, and one SNP that reduced substrate affinity. The objective of the current study is to investigate functional actions of the NAT1 coding exon SNPs for ABP N-acetyltransferase and the N-OH-ABP O-acetyltransferase and to interpret these results in the context of cancer risk following ABP exposures.

## Methods

### Expression of NAT1 Reference and NAT1 Alleles Possessing Individual SNPs

The human NAT1 coding exon was amplified by polymerase chain reaction (PCR) using plasmid pESP-3 containing NAT1*4 and other NAT1 SNPs as previously described (Zhu and Hein, 2008). COS-1 cells (SV40-transformed African green monkey kidney cells) were purchased from American Type Culture Collection (Manassas, VA United States) and cultured as previously described (Zhu and Hein, 2008). As described more fully previously (Zhu and Hein, 2008), constructed plasmids were transiently transfected using Lipofectamine and Plus reagent, 10 μg of either NAT1*4 (no SNP), a mock empty vector, or NAT1 possessing a single SNP containing vector pCR3.1 and 80 ng pCMV SPORT-βgal (Invitrogen) plasmid. Transfected cells were harvested after 48 h and then removed, washed, and pelleted by centrifugation. Cell pellets were disrupted in lysis buffer (20 mM sodium phosphate, pH 7.4, 1 mM EDTA, 1 mM dithiothreitol, 10 μM phenylmethanesulfonyl fluoride, 10 μM leupetin, 1 μM pepstatin A, 4 μM aprotinin) by sonication on ice. Cell debris was precipitated at 15,000 X g for 15 min at 4°C. No NAT1 activity was detectable in mock-transfected cells. To account for possible differences in transfection efficiency, COS-1 cell lysates were assayed for β-galactosidase activity with O-nitrophenyl β-D-galactopyranoside (Zhu and Hein, 2008).

### ABP N-Acetyltransferase Assays

ABP N-acetyltransferase reactions were conducted as previously described (Hein et al., 2006). Briefly, 50 μL cell lysate (buffer composition described above), ABP (0–1 mM) and acetyl coenzyme A (1 mM) were incubated at 37°C for 10 min. HPLC separation of ABP and N-acetyl-ABP was achieved using a gradient of 85:15 sodium perchlorate pH 2.5: acetonitrile to 35:65 sodium perchlorate pH 2.5: acetonitrile over 10 min and was quantitated by absorbance at 260 nm. Mock transfected COS-1 cells were used as a negative control. Transiently transfected cells were normalized to total protein and β-galactosidase activity was used to normalize for transfection efficiency as previously described (Zhu and Hein, 2008). Protein concentrations were determined with the Bio-Rad protein assay (Bradford, 1976).

### N-OH-ABP O-Acetyltransferase Assays

N-OH-ABP O-acetyltransferase assays were conducted as previously described (Hein et al., 2006). Briefly assays containing 100 μg cell lysate in buffer described above), 1 mM acetyl coenzyme A, 1 mg/ml deoxyguanosine (dG), and 0–1 mM N-OH-ABP were incubated at 37°C for 10 min. HPLC separation was achieved using a gradient of 80:20 sodium perchlorate pH 2.5: acetonitrile to 50:50 sodium perchlorate pH 2.5: acetonitrile over 3 min and dG-C8-ABP adduct was detected at 300 nm. Transiently transfected cells were normalized to total protein and β-galactosidase activity was used to normalize for transfection efficiency as previously described (Zhu and Hein, 2008). Protein concentrations were determined with the Bio-Rad protein assay (Bradford, 1976).

### Data Analysis

ABP N-acetyltransferase and N-OH-ABP O-acetyltransferases activities carried out at various substrate concentrations were subjected to Michaelis-Menten kinetic analysis and the apparent Km and Vmax were calculated using the Michaelis-Menten program in Graphpad Prism (San Diego, CA). Differences in apparent Km and Vmax between reference allele NAT1*4 and SNP variants were tested for significance using one-way ANOVA followed the Dunnett multiple comparison tests.

## Results

**FIGURE 1:**
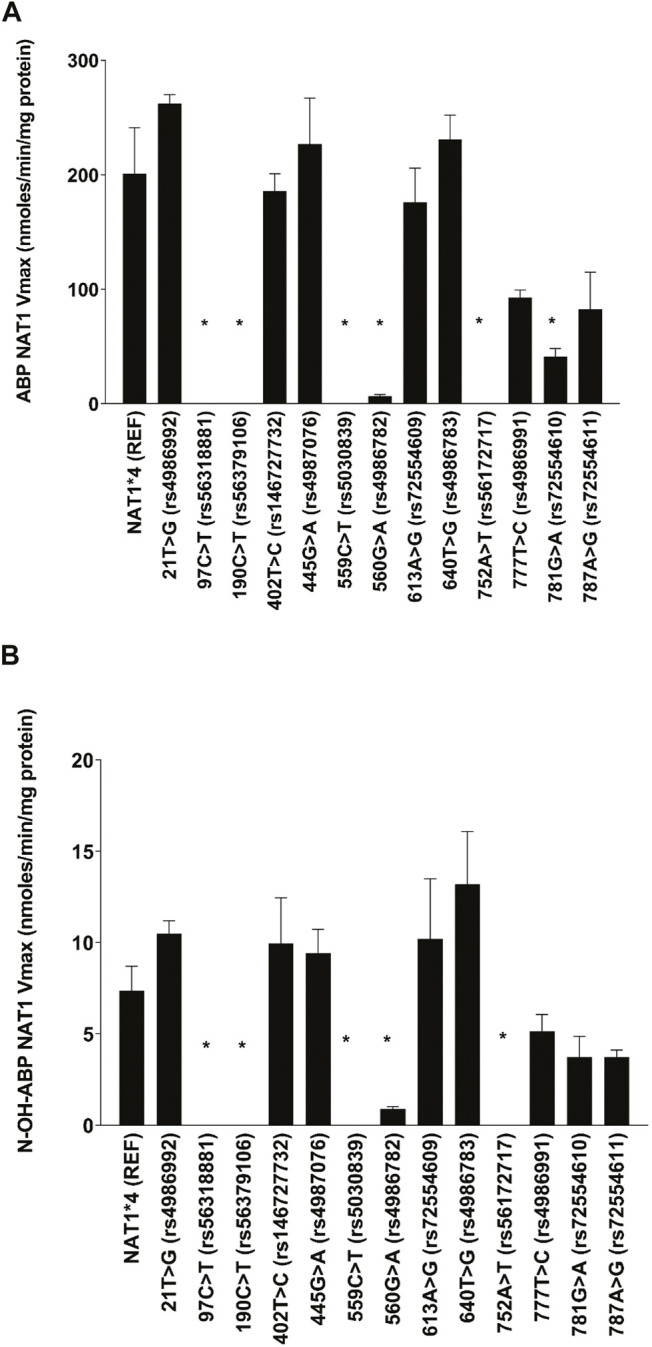
Apparent Vmax for **(A)** ABP N-acetylation and **(B)** N-OH-ABP O-acetylation for NAT1*4 (reference) and *NAT1* variants possessing the SNP labeled on the abscissa. Each bar represents Mean ± SEM from three transfections in duplicate. *Significantly less than NAT1*4 for ABP-N-acetylation (*p* ≤ 0.001) and N-hydroxy-ABP O-acetylation (*p* < 0.05).

The effects of individual single nucleotide polymorphisms (SNPs) in the NAT1 coding exon on Michaelis-Menten kinetic constants was assessed for ABP N-acetyltransferase and N-OH-ABP O-acetyltransferase catalyzed by recombinant human NAT1 expressed in COS-1 cells. As shown in Figure 1, NAT1 coding region SNPs 97C > T (rs56318881) (R33stop), 190C > T (rs56379106) (R64W), 559C > T (rs5030839) (R187stop) and 752A > T (rs56172717) (D251V) reduced ABP N- acetyltransferase and N-OH-ABP O-acetyltransferase activity below detection. 21T > G (rs4986992) (synonymous), 402T > C (rs146727732) (synonymous), 445G > A (rs4987076) (V149I), 613A > G (rs72554609) (M205V) and 640T > G (rs4986783) (S241A) did not significantly affect Vmax for ABP N-acetyltransferase or N-OH-ABP O-acetyltransferase. 781G > A (rs72554610) (E261K), and 787A > G (rs72554611) (I263V) slightly decreased ABP N-acetyltransferase and N-OH-ABP O-acetyltransferase whereas 560G > A (rs4986782) (R187Q) substantially and significantly reduced them.

**FIGURE 2:**
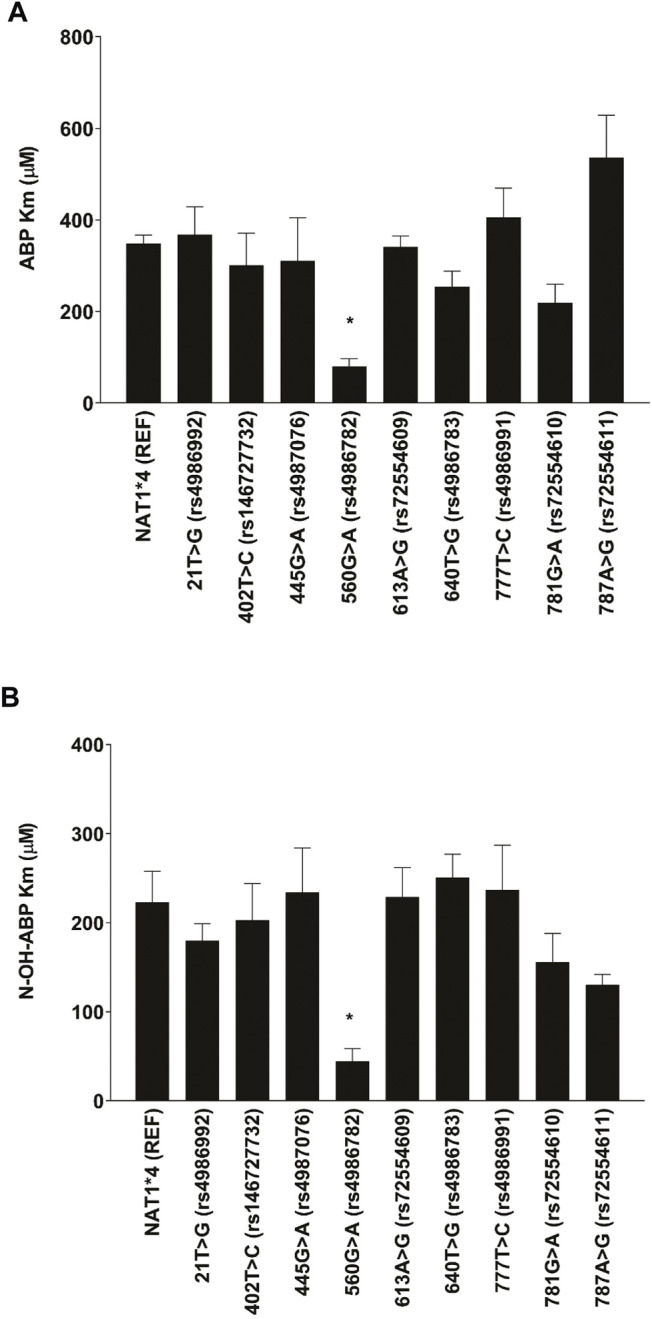
Apparent Km for **(A)** ABP and **(B)** N-OH-ABP for NAT1*4 (reference) and *NAT1* variants possessing the SNP labeled on the abscissa. Each bar represents Mean ± SEM from three transfections in duplicate. *Significantly less than NAT1*4 for ABP (*p* < 0.05) and N-OH-ABP (*p* < 0.01).

As shown in Figure 2, 560G > A (rs4986782) (R187Q) significantly reduced the apparent Km for ABP and N-OH-ABP a finding that was not observed with any of the other NAT1 SNPs tested. None of the other NAT1 SNPs significantly (*p* > 0.05) affected apparent Km towards ABP or N-OH-ABP.

## Discussion

The frequency of the various NAT1 SNPs varies with ethnicity (https://www.ncbi.nlm.nih.gov/snp/rs4986782?vertical_tab=true#frequency_tab). 560G > A (rs4986782) (R187Q) is the most frequent SNP in the NAT1 coding exon with a global frequency of 1.75% but a 13-fold higher frequency (23.8%) has been reported in Lebanese resulting in nearly 50% of a Lebanese population heterozygous for the allele (Dhaini and Levy, 2000).

The effects of SNPs in the human NAT1 coding exon on N-acetyltransferase and O-acetyltransferase activities have previously been reported following recombinant expression in bacteria (Doll et al., 1997; Butcher et al., 1998; Hughes et al., 1998; Payton and Sim, 1998), yeast (Fretland et al., 2001; Fretland et al., 2002) COS-1 cells (Zhu and Hein, 2008; Zhu et al., 2011) and/or CHO cells (Millner et al., 2012). Our findings that NAT1 coding region SNPs 97C > T (rs56318881) (R33stop), 190C > T (rs56379106) (R64W), 559C > T (rs5030839) (R187stop) and 752A > T (rs56172717) (D251V) reduced ABP N- acetyltransferase and N-OH-ABP O-acetyltransferase activities below detection is consistent with previous reports on *p*-aminobenzoic acid N-acetyltransferase activity, NAT1-selective substrate, in the expression systems described above. 781G > A (rs72554610) (E261K), and 787A > G (rs72554611) (I263V) caused slight but significant reduction in ABP N-acetyltransferase and of N-OH-ABP O-acetyltransferase activities that likely is relatively unimportant in risk assessment. Previous studies following recombinant expression of NAT1 in yeast reported that 781G > A (rs72554610) (E261K) caused slight reductions in N-acetylation of the arylamine carcinogens 2-aminofluorene (Fretland et al., 2001) and ABP (Fretland et al., 2002) and the O-acetylation of N-hydroxy-2-aminofluorene (Fretland et al., 2002).

560G > A (rs4986782) (R187Q) was identified as a reduced N-acetylation phenotype based upon its effects on the N-acetylation of *p*-aminosalicylic acid in human subjects (Hughes et al., 1998). In the present study 560G > A (rs4986782) (R187Q) substantially reduced both ABP N-acetyltransferase and N-OH-ABP O-acetyltransferase, consistent with the identification of NAT1*14A and NAT1*14B as reduced function alleles. As previously reviewed (Hein, 2009), the R187 is partially exposed to the protein surface and the active site and forms hydrogen bonds with E182, K188, and T289. These interactions help shape the active site pocket and R187Q may cause partial loss of these interactions leading to destabilization of NAT1 consistent with reduced expression of human NAT1 protein following recombinant expression in yeast (Fretland et al., 2001) and COS-1 cells (Zhu and Hein, 2008). R187Q also may cause changes in the conformation and/or dynamics of the active site or protein structure and thus alter substrate selectivity and catalytic activity.

Previous studies reported that the G560A(R187Q) SNP caused a 6–10-fold *decrease* in the affinity of human recombinant NAT1 for *p*-aminobenzoic acid (Zhu and Hein, 2008; Millner et al., 2012). Following administration of *p*-aminosalicylic acid, individuals with the NAT1*14A allele possessing 560G > A (rs4986782) (R187Q) exhibit higher maximum concentrations and overall area under the plasma level versus time curve drug levels irrespective of dosing regimen (Sy et al., 2015), consistent with NAT1*14s designation as a reduced function allele (Hughes et al., 1998; Fretland et al., 2001; Fretland et al., 2002; Zhu and Hein, 2008; Hein et al., 2018).

NAT1 is a major pathway in the urinary bladder mucosa for the bioactivation of urinary N-hydroxy arylamines to reactive N-acetoxy esters that form DNA adducts (Badawi et al., 1995). The frequency of 560G > A (rs4986782) (R187Q) varies with ethnic origin but it was identified in almost half of a Lebanese population (Dhaini and Levy, 2000). Notably, the incidence of urinary bladder cancer has increased markedly in Lebanon and is currently the second most incident cancer among males (Shamseddine et al., 2004). The frequency of 560G > A (rs4986782) (R187Q) is 7-fold higher among urinary bladder cases than controls in Lebanon (Yassine et al., 2012) and is significantly associated with higher muscle-invasiveness and higher tumor grade of urinary bladder tumors (El Kawak et al., 2020). In the current study 560G > A (rs4986782) (R187Q) caused a 4 to 5-fold *increase* (*p* < 0.05) in affinity for both ABP and N-OH-ABP. None of the SNPs in the NAT1 coding exon affect V93, K100, I106, F125, L209, S215, V216 or F217 residues in the active site that may be important for ABP access (Zhou et al., 2013). The increase in affinity for ABP and N-OH-ABP is consistent with an increased risk for urinary bladder cancer following lower levels of exposure. In contrast, higher activity NAT1 alleles such as NAT1*10 (Hein et al., 2018) are associated with lower prevalence of urinary bladder cancer (Cascorbi et al., 2001).

560G > A (rs4986782) (R187Q) also has been associated with higher prevalence of lung cancer in cigarette smokers (Bouchardy et al., 1998). In a subsequent study, the association of NAT1*14 alleles possessing 560G > A (rs4986782) (R187Q) with higher incidence of lung cancer nearly reached statistical significance while an inverse association with the NAT1*10 allele achieved statistical significance (McKay et al., 2008). Thus the results with lung cancer incidence in smokers are similar to those for urinary bladder cancer incidence described above.

In conclusion, the current study confirms and expands upon previous studies that have characterized the effects of SNPs in the NAT1 coding exon on N-acetylation of ABP and O-acetylation of N-OH-ABP. Our findings confirm previous studies reporting that NAT1*14 alleles are reduced function alleles, but in contrast to *p*-aminobenzoic acid, the current study showed that the 560G > A (rs4986782) (R187Q) caused a 4 to 5-fold *increase* (*p* < 0.05) in affinity for both ABP and N-OH-ABP. The increase in affinity for ABP and N-OH-ABP shows that in altering the site for substrate binding, 560G > A (rs4986782) (R187Q) can cause opposite effects on substrate affinity. The findings are consistent with an increased risk for urinary bladder cancer and perhaps also lung cancer in individuals possessing NAT1*14 alleles following lower levels of exposure to ABP as would be expected in active and particularly passive exposure following use of cigarette and other tobacco products.

## Data Availability

The original contributions presented in the study are included in the article/Supplementary Material, further inquiries can be directed to the corresponding author.
